# Primary Chordoma of the Nasopharynx: A Rare Case Report and Review of the Literatures

**DOI:** 10.1155/2019/3826521

**Published:** 2019-09-23

**Authors:** Ery Kus Dwianingsih, Yosinta Snak, Hanggoro Tri Rinonce, Brian Wasita, Ester Lianawati Antoro, Samir S. Amr

**Affiliations:** ^1^Department of Anatomical Pathology, Faculty of Medicine Public Health and Nursing, Universitas Gadjah Mada, Yogyakarta, Indonesia; ^2^Department of Anatomical Pathology, Faculty of Medicine, Universitas Sebelas Maret, Solo, Indonesia; ^3^Department of Pathology and Laboratory Medicine, Istishari Hospital, Amman, Jordan

## Abstract

Primary chordoma of the nasopharynx is an extremely rare malignant tumor of notochordal origin in the extra-osseous axial skeleton. It presents as a soft tissue mass without involvement of the skull base bone (clivus) and may mimic other lesions of the nasopharynx. A 26-year-old male patient is presented with nasal obstruction and congestion for the last 3 years. Physical and radiological examination revealed a mass in the naso-oropharyngeal region. It was suspected to be a cystic mass or abscess on radiological imaging. However, histopathological examination revealed a chordoma. We review all 20 cases of primary nasopharyngeal chordoma reported previously in the literature. Nasopharyngeal chordoma should be considered in the differential diagnosis of nasopharyngeal mass due to its unspecific appearance on clinical and radiology examination.

## 1. Introduction

Chordoma is a rare malignant bone tumor that occurs in any site along the course of embryogenic notochord and typically in the axial skeleton [1]. It affects males twice compared to females, and the age range is 40–60 years [1, 2]. The topographic distribution of chordoma includes sacrococcygeal (50%), cranio-cervical/spheno-occipital (35%), and thoraco-lumbar spine (15%). Cranio-cervical chordomas most often involve the dorsum sellae, the clivus, and rarely the nasopharynx [2]. Unusual locations had been reported including the mandible and the maxilla (dental chordoma), some also with nasal and paranasal presentations [3].

Histologically, the hallmark of chordoma is the presence of large physaliphorous cells (Greek: *physalis*: bubbles) with vesicular nuclei embedded in a homogenous, intercellular substance. The vacuoles contain either mucinous substance or glycogen. The intercellular substance is considered as partly the result of cell secretion and in part of products of cell degeneration [4].

Chordoma is categorized into 3 types: Classical or conventional chordoma, which is rich in physaliphorous cells, and is the most frequent type of chordoma; chondroid chordoma, resembling both chordoma and chondrosarcoma; and dedifferentiated chordoma featuring sarcomatous areas which are comprised of spindle-shaped and polygonal cells, and is the most aggressive type of chordoma [5].

Immunohistochemically, the notochordal cells strongly express cytokeratin but weakly vimentin in the earlier developmental stages of the foetus. In later developmental stages, it shows distinct increase in vimentin expression and a slight decrease in cytokeratin expression [6]. Chordoma may also express epithelial membrane antigen (EMA), carcinoembryonic antigen (CEA), S100 protein, alpha 1-antichymotrypsin, and lysozyme [7]. Brachyury had been proposed as the most specific diagnostic marker of chordoma's neoplastic cells since it is a transcription factor which is required for notochord development during embryogenesis [8].

Herein we report a case of primary chordoma of the nasopharynx, its characteristics, and a review of previously reported cases.

## 2. Case Presentation

A 26-year-old man was admitted to the ear, nose, and throat department of our hospital with nasal obstruction and congestion of 3 years duration. There was no history of headache, epistaxis, visual changes, or cranial nerve palsy/paresis.

**Figure 1:**
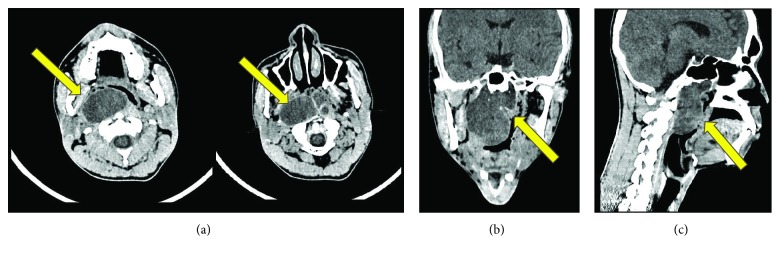
Axial (a), Coronal (b), and Sagittal (c) MSCT showed a round large lobulated mass in naso-oropharyngeal area, with well-defined margin (arrow) without involvement of the clivus.

Physical examination showed a mass in naso-oropharyngeal region with anterior displacement of the uvula. Radiological examination by axial, coronal, and sagittal MSCT with and without contrast confirmed the presence of a round-large lobulated mass in naso-oropharyngeal region, approximately 5 × 6 cm with well-defined margin without involvement of the clivus (Figure 1). It was interpreted as a naso-oropharyngeal cystic mass, possibly a naso-oropharyngeal abscess. Chest X-ray was unremarkable. Laboratory workup revealed that all haematological and biochemical parameters were within normal limits.

Biopsies were taken from different parts of the mass (right and left nasopharynx and oropharynx) but they were superficial and did not reach the tumor itself. Histologically, they showed nonspecific chronic inflammation. The patient was treated with antibiotic and anti-inflammatory drugs, without any clinical improvement.

**Figure 2:**
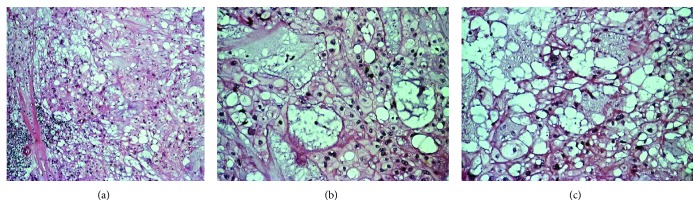
Cords and lobules of physaliferous cells separated by fibrous septa [(a), H & E stain, 100x magnification], abundant eosinophilic and vacuolated cytoplasm with prominent vesicular nuclei embedded in extensive myxoid stroma. [(b, c), H & E stain, 200x magnification].

A deep biopsy from the mass was eventually obtained, and it revealed a chordoma of the nasopharynx. The patient underwent surgery to excise the mass with trans-palatal rhinotomy approach, under general anaesthesia. Approximately 30 cc of fragmented tissue was received. Macroscopically, the tumor tissue was firm and lobulated, some was covered by brownish white mucosa. Microscopic examination showed cords and lobules of physaliphorous cells embedded within extensive myxoid stroma, separated by fibrous septa. Some of tumor cells already invaded adjacent skeletal muscle. The physaliphorous cells were variable in size, with abundant eosinophilic to clear cytoplasm, some were vacuolated, with prominent vesicular nuclei. Other tumor cells were small with pyknotic nuclei. Mitotic figures were inconspicuous (Figure 2).

**Figure 3:**
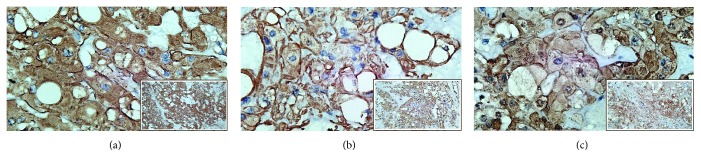
Immunohistochemistry analysis with 3 different markers (400x magnification) showed strong cytoplasmic expression of Cytokeratine (a), which was as strong as Vimentin (b), Meanwhile, S100 demonstrated a slightly weaker expression (c), (inset : 200x magnification).

The immunohistochemical studies revealed positive cytoplasmic staining for cytokeratin and vimentin. Meanwhile, expression of S100 was slightly weaker (Figure 3). Immunostaining of brachyury was not performed since it was not available in any of the pathology laboratories in our country. After surgery, the patient showed excellent recovery with no other complication. The patient had been closely followed up at the ENT outpatient clinic.

## 3. Discussion

Chordoma is a rare malignant bone tumor, primarily involving both ends of the axial skeleton, and arise from the remnant of the notochord. It was first described by Virchow in 1857 as a tumor made up of vacuolated or physaliphorous cells derived from rests of embryonic notochord along the midline central nervous system axis [9].

The notochord is a rod-like aggregate of cells extending the entire length of the embryo on the midline, ventral to the developing neural tube. The embryonic notochord degenerates early in fetal development after being surrounded by sclerotome mesenchymal cells and remains as the nucleus pulposus within intervertebral disc. However, in some cases residual notochord cells remain outside the inter-vertebral disc and may become neoplastic [10].

Primary nasopharyngeal chordoma arises in the extraosseous nasopharyngeal soft tissues and may or may not have a smaller intraosseous component along the course of the medial basal canal. The medial basal canal is considered the cephalad exit tract of the notochord as it moves from its intraclival location ventrally into the midline nasopharyngeal soft tissues [3]. Chordomas arising from the skull base/clivus with extension into the nasopharynx area are excluded in the list of primary nasopharyngeal chordomas [1]. They are preferably diagnosed as clival chordomas presenting as nasopharyngeal mass [11, 12].

To the best of our knowledge, only 20 cases of primary nasopharyngeal chordoma had been previously reported, with 5 of them presented without any bone involvement [1, 3, 13–19]. We summarize the clinical, radiological, and histological features of patients with nasopharyngeal chordoma in Table 1, including the current case.

Primary nasopharyngeal chordoma commonly occurs in young adults with an average age of 39.6 years, ranging from 8 to 80 years. It affects males (57.1%) more than females (42.8%) with a male to female ratio of 1.3 : 1. The main clinical presentation is nasal obstruction, reported in 16 cases (76.2%). Other symptoms include hearing impairment, headache, difficulty in swallowing, dryness of the mouth, nasal bleeding, diplopia, numbness, dropping of upper eye lid, difficulty in breathing, and nasal speech. Almost all patients with primary nasopharyngeal chordoma show unremarkable laboratory findings and normal general physical examination. The tumor size ranged from 2.5 to 8.2 cm and all were lobulated. Chondroid chordoma was found in a single case with progressive manifestation within 3 months prior to diagnosis, and chondrosarcoma cannot be excluded in the differential diagnosis. Twenty cases were identified as classical chordoma (95.2%) and 6 cases (28.6%) showed slow growing tumors, with clinical manifestation observed within 6 months up to 5 years prior to the diagnosis. 5 cases had no further information regarding the onset of their clinical manifestation.

Histopathologically, 20 cases (including the current case) were classical chordomas showing numerous physaliphorous cells arranged in cord or lobules with myxoid stroma separated by fibrous tissue, meanwhile 1 case was chondroid variant, showing chondroid differentiation resembling chondrosarcoma [5]. Radiologically, classical and chondroid chordoma can be misdiagnosed with cyst, abscess, nasopharyngeal angiofibroma, or even nasopharyngeal carcinoma. Clinically, unlike malignant lesion, benign lesion do not progress in size in a short time, or invade or destroy the surrounding tissues. Cystic lesions are identified by single or multiple spaces lined with cuboidal or flattened epithelial cells [20]. Nasopharyngeal abscess formation is characterized by prominent necrotic area, infiltrated by numerous inflammation cells, specially leucocytes [21]. Angiofibroma showed proliferation of fibroblasts along with numerous blood vessels [22]. Lastly, nasopharyngeal carcinoma is characterized by islands of epithelial cells which consisted of round, oval to spindle atypical cells, infiltrating to surrounding area [23]. Up to now, dedifferentiated type of primary nasopharyngeal chordoma has not been reported yet.

In all cases of reported nasopharyngeal chordomas, metastasis was not observed. However, there had been reports of cases of chordoma that metastasized to the peritoneum, pleura [24], liver [1, 24], lung, bone, soft tissue, skin [1, 2, 24], and lymph node [12].

This case report was interesting on several counts. We experienced some difficulties during the pre-operative diagnosis due to the nonspecific features of chordoma on clinical and radiological examinations. Moreover, particular location of the tumor was difficult to be reached and biopsied. Those factors resulted in delayed accurate diagnosis. Because of the location in nasopharynx region, the differential diagnosis of chordoma should be made from other nasopharyngeal masses. Although CT and MRI features are nonspecific, they may be suggestive of chordoma including midline location, expansible or lobular soft tissue mass with well-defined margin. Other nasopharyngeal malignancies may destroy clival bone but do not demonstrate this midline tract. CT is ideal for evaluating the bony involvement, whereas MRI is useful in evaluating the surrounding soft tissues and extension into adjacent structures [3]. Chordoma is considered to have poor sensitivity of radiotherapy and chemotherapy; however, surgery was shown as the treatment of choice [19, 24].

## 4. Conclusion

Primary chordoma of the nasopharynx is a rare but an important lesion to be considered when a midline nasopharyngeal mass is found with or without involvement of clival sinus tract. It has no specific appearance on clinical and radiological examination. Obtaining the right biopsy material is required for proper histopathological diagnosis of the tumor.

## Figures and Tables

**Table 1:** 

No	References	Age/Sex	Chief complaint	Radiology	Bony involvement	Differential diagnosis	Tumor size (cm)	Gross	Pathology
1	Handosa Bey, 1949 [13]	16/F	Nasal obstruction for 2.5 years, headache	Mass in the skull base (sphenoid-base), filling nasophryngeal cavity	Bony trabecula involvement	Osteoclastoma	N/A	Soft friable tissue	Classic type
2	Seltzer et al., 1961 [14]	24/F	Six months of difficulty in swallowing, strangling sensation, moderate pain localized in the back of the throat, nasal speech	Large lobular and tender mass over the left nasopharyngeal floor	No erotion, nor other involvement of bone	Retropharyngeal abcess, infected cyst of the pharynx	4 × 4	Lobulated, soft, whitish mass with a cleft in the center	Classic type
3	Hingorani et al., 1970 [15]	62/M	Three months of progresive left-sided nasal obstruction	Large nasopharyngeal mass sitting on the sphenoidal sinus	Some bony spiculation and erosion	Chondrosarcoma cannot be positively excluded	N/A	Lobulated, fleshy, friable, brownish grey	Chondroid type
4	Maru et al., 1988 [16]	52/M	Nine months of nasal obstruction, bilateral hearing impairment	A huge soft tissue mass completely occupying the nasoppharyrtx and protruding into the orophaxynx	No bone involvement	Chondroma, Chondrosarcoma	8 × 6	A globular smooth mass	Classic type
5	Maru et al., 1988 [16]	20/M	Painful swelling of the right side of the face for 4 months	Soft-tissue mass in the right maxillary antrum with bone destruction of the medial, and anterolateral walls and roof	Involving anterolateral wall ofthe right maxilla, alveolar margin, gingivo-buccal sulcus and hard palate	chondrosarcoma	N/A	Soft-tissue mass with bone destruction	Classic type
6	Boyle et al., 1954 [17]	43/M	Double vision for 12 months, nasal obstruction for 3 months, and severe periotbital headaches and deafness	Mass instilled into the nose until sphenoid sinus	Bone destruction at the base of the skull	Nasopharyngeal carcinoma, adenocarcinoma	N/A	Hard and smooth lobulated tumour in a mucinous substance	Classic type
7	Hampal et al., 1992 [18]	80/F	Six months of dysphagia for solids and weight loss	Well circumscribed mass in the left parapharyngeal space	Absence of bony/spine involvement	Nasopharyngeal carcinoma	4 × 4	Greyish-white with a lobulated surface and gelatinous on the cut surface	Classic type
8	Wright et al., 1967 [19]	16/M	Complete bilateral nasal obstruction and nose bleeds for several months	Soft tissue swelling filling the nasopharynx	No bone involvement	Nasopharyngeal carcinoma	8 × 2	Swelling mass	Classic type
9	Wright et al., 1967 [19]	26/F	Increasing bilateral nasal obstruction	Large soft tissue mass filling the nasopharynx	No bone involvement	Nasopharyngeal carcinoma	N/A	Large tumor mass	Classic type
10	Wright et al., 1967 [19]	53/F	Right-sided headaches for 8 months, diplopia, numbness of the right cheek, tongue and gums, tinnitus, and deafness on the right side	Mass in the nasopharynx with erosion part of the right middle cranial fossa and pituitary fossa	Extension of erosion into the left middle cranial fossa and petrous apex	Nasopharyngeal carcinoma	N/A	Large tumor mass	Classic type
11	Wright et al., 1967 [19]	52/M	Left frontal pain and a drooping upper eyelid	Raised intracranial pressure with erosion of the pituitary fossa and destruction of the sphenoid and adjacent left ethmoid cells	Destruction of sphenoid, ethmoid and pituitary fossa	Nasopharyngeal carcinoma	N/A	Firm blue cyctic swelling	Classic type
12	Nguyen et al., 2009 [3]	8/F	Nasal obstruction, dryness of the mouth, difficulty in breathing,	Midline lobular and expansile mass with internal septation centered in the nasopharynx	Bony lytic changes along the anterior surface of the clivus (5/5)	Nasopharyngeal carcinoma, NonHodgkin lymphoma	N/A	Lobulated	Classic type
13	Nguyen et al., 2009 [3]	65/F							
14	Nguyen et al., 2009 [3]	56/M							
15	Nguyen et al., 2009 [3]	53/M							
16	Nguyen et al., 2009 [3]	32/F							
17	Yan et al., 2010 [1]	13/M	One to five years of nasal obstruction, headache and hearing loss (2/4)	Lobular and expansile nasopharyngeal mass with irregular intra tumor calsification	No bony involvement into the clivus	Nasopharyngeal Carcinoma, Juvenile Nasopharyngeal Angiofibroma	2.5–8.2	Lobulated, well defined with intra tumor septa	Classic type
18	Yan et al., 2010 [1]	31/M							
19	Yan et al., 2010 [1]	38/M							
20	Yan et al., 2010 [1]	66/F							
21	Present case	26/M	Three years of nasal obstruction and congestion	Lobulated naso-oropharyngeal mass with displacement of uvula anteriorly	No bony involvement into the clivus	Naso-oropharyngeal cystic mass/abcess.	5 × 6	Lobulated, brownish white, some parts with black spot	Classic type

N/A = Not Available
